# A preliminary account of the fly fauna in Jabal Shada al-A’la Nature Reserve, Saudi Arabia, with new records and biogeographical remarks (Diptera, Insecta)

**DOI:** 10.3897/zookeys.636.9905

**Published:** 2016-11-24

**Authors:** Magdi S. El-Hawagry, Mahmoud S. Abdel-Dayem, Ali A. Elgharbawy, Hathal M. Al Dhafer

**Affiliations:** 1 Entomology Department, Faculty of Science, Cairo University, Giza 12613, Egypt; 2 Plant Protection Department, College of Food and Agriculture Sciences, King Saud University, Riyadh 11451, PO Box 2460, Kingdom of Saudi Arabia

**Keywords:** Afrotropical, Al-Baha Province, Al-Sarah, Al-Sarawat Mountains, Arabian Peninsula, Eremic Zone, fly species, new records, Palaearctic, Tihama

## Abstract

The first list of insects of Al-Baha Province, Kingdom of Saudi Arabia (KSA) was published in 2013 and contained a total of 582 species; an addendum to this list was published in 2015 adding 142 species and bringing the total number recorded from the province to 724 insect species representing 17 orders. The previous two studies excluded Jabal Shada al-A’la Nature Reserve (SANR), so the present study in SANR, as belonging to Al-Baha Province, are complementary to the previous two. The present study presents a preliminary list of Diptera (Insecta) in SANR, with remarks on their zoogeography, and is the first of a series of planned ecological and systematic studies on different insect orders as one of the outputs of a project proposed to study the entire insect fauna of SANR.

A total number of 119 Diptera species belonging to 87 genera, 31 tribes, 42 subfamilies, and representing 30 families has been recorded from SANR in the present study. Some species have been identified only to the genus level and listed herein only because this is the first time to record their genera in KSA. Fourteen of the species are recorded for the first time for KSA, namely: *Forcipomyia sahariensis* Kieffer, 1923 [Ceratopogonidae]; *Chaetosciara* sp. [Sciaridae]; *Neolophonotus* sp.1; *Neolophonotus* sp.2; *Promachus sinaiticus* Efflatoun, 1934; *Saropogon longicornis* (Macquart, 1838); *Saropogon* sp. [Asilidae]; *Spogostylum tripunctatum* (Pallas *in* Wiedemann, 1818) [Bombyliidae]; *Phycus* sp. [Therevidae]; *Hemeromyia* sp.; *Meoneura palaestinensis* Hennig, 1937 [Carnidae]; *Desmometopa inaurata* Lamb, 1914 [Milichiidae]; *Stomoxys niger* Macquart, 1851 [Muscidae]; and *Sarcophaga palestinensis* (Lehrer, 1998) [Sarcophagidae].

Zoogeographic affinities of recorded fly species suggest a closer affiliation to the Afrotropical region (46%) than to the Palearctic region (23.5%) or the Oriental region (2.5%). This supports the previous studies’ conclusions and emphasizes the fact that parts of the Arabian Peninsula, including Al-Baha Province, ought to be a part of the Afrotropical Region rather than of the Palaearctic Region or the Eremic Zone.

## Introduction

**Figure 1.**
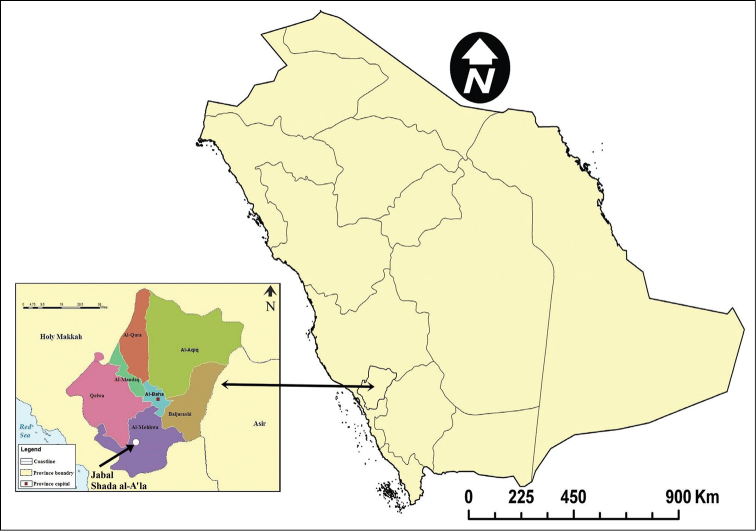
Map of Saudi Arabia showing Al-Baha Province and Jabal Shada al-A‘la Nature Reserve.

Al-Baha Province (Fig. 1) is situated in the south-western part of the Kingdom of Saudi-Arabia (KSA) between the Holy Makkah and Asir provinces. It is the smallest province in KSA (approximately 10,362 km^2^), situated at 41–42 °E and 19–20 °N. It is characterized by natural tree cover (El-Juhany and Aref 2013) and agricultural plateaus. Huge and steep rocky mountains divide the province into two main sectors, a mountainous area known as ‘Al-Sarat’ or ‘Al-Sarah’ with an elevation of 1500–2450 m above sea level at the east forming a part of Al-Sarawat Mountains range, and a lowland coastal plain in the west, known as ‘Tihama’. The second sector, Tihama, is divided into two districts, Al-Mekhwa and Qelwa (Alahmed et al. 2010, El-Hawagry et al. 2013, 2015). Jabal Shada al-A’la Nature Reserve (SANR) lies between latitudes 19.8149N–19.8763N and longitudes 41.2855E–41.3501E (Fig. 1). It is an isolated granite mountain massif made up of jagged spires and pinnacles, located in Al-Mekhwa district, 20 km to the south-west of Al-Mekhwa city, the capital of the district. It is a dissonant of the Sarawat Escarpment in the foothills of Tihama, measuring 68.62 square kilometers. Its location and its altitudinal range from 490 to 2,222 meters above sea level ensures high rainfall, a wide range of micro-climates, and a high level of biological diversity (SWA 2016).

**Figure 2.**
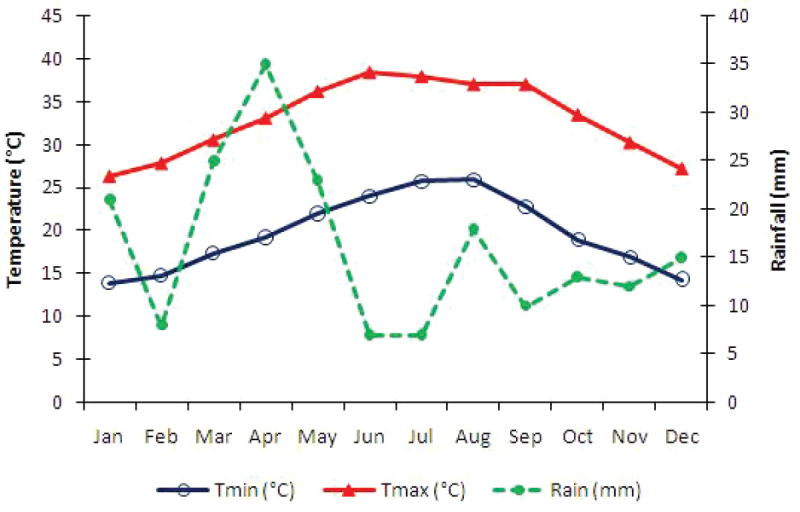
Monthly average temperatures and rainfall in 50 years (1950–2000). In Jabal Shada al-A‘la Nature Reserve (Worldclim database: http://www.worldclim.org/).

In the lowland coastal plain, Tihama, the climate is hot in summer, warm in spring and mild in winter, with less than 100 mm of annual rainfall. In the mountainous area, Al-Sarah, the weather is generally cooler due to high altitude, in addition to the formation of clouds and fog accompanied by thunderstorms in winter, with a rainfall average of 405 mm annually (Ibrahim and Abdoon 2005; El-Hawagry and Al Dhafer 2015). The climate in SANR is intermediate between the climates in these two sectors, with a rainfall average of approximately 200 mm annually (Fig. 2).

SANR, as an isolated mountain massif, supports an exceptionally rich flora; with approximately 500 plant species recorded, including 63 key plant taxa including endemics and Afrotropical relicts, it is the site of highest botanical diversity known in Saudi Arabia. The exceptional floral diversity of SANR, together with the presence of griffon vultures and endemic birds of the southwestern mountains and carnivores such as, the Arabian red fox [*Vulpes vulpes arabica* Thomas, 1902], Arabian caracal [*Caracal caracal schmitzi* (Matschie, 1912)], striped hyaena [*Hyaena hyaena sultana* (Pocock, 1934)], Arabian wolf [*Canis lupus arabs* Pocock, 1934], sand cat [*Felis margarita harrisoni* Hemmer, Grubb & Groves, 1976], and reportedly the Arabian leopard [*Panthera pardus nimr* Hemprich & Ehrenberg, 1833], makes this small protected area a unique treasure of biological diversity. Small communities on the mountain grow a distinctive variety of coffee and other crops in terraced fields (El-Hawagry et al. 2013; SWA 2016; UAEinteract 2016).

The purpose of this paper is to present a preliminary list of Diptera (Insecta) in SANR, Al-Baha Province, KSA, with remarks on their zoogeography. This is not the final list of Diptera that occur at SANR with the study serving as a basis for further investigations as many additional collected species are still unidentified and further studies are planned to be carried out at SANR. Also, this is the first of a series of planned ecological and systematic studies on different insect orders as one of the outputs of a project proposed to study the entire insect fauna of SANR.

El-Hawagry et al. (2013, 2015) studied the insect fauna of Al-Baha Province excluding SANR, so the present study and other future studies in SANR are complementary to the previous two studies. Studies on the fauna of SANR are of particular interest as this area lies in a part of the Arabian Peninsula which is thought by many authors to touch three of the main zoogeographical regions: the Palaearctic, the Afrotropical, and the Oriental (Hölzel 1998).

The Afrotropical Region is supposed to encompass all of Africa south of the Sahara, with the island of Madagascar and the nearby smaller islands. Many authors add parts of the Arabian Peninsula to the Afrotropical Region as well, but there seems to be no agreement as to how much (El-Hawagry et al. 2015). This may be deduced from the fact that the south-western and southern parts of the Arabian Peninsula including Al-Baha Province are strongly influenced by a subtropical to tropical climate with spring and summer rains (Abdullah and Al-Masroui 1998), and are thus dominated by a xeromesic tropical flora of palaeotropical origin, that in fact represents the impoverished northern part of an African flora (Ghazanfar and Fisher 1998; Hegazy et al. 1998). Examples of plant species with this conspicuous distribution pattern, linking south-west Arabia with the other side of the Red Sea, and commonly represented in SANR are: *Barleria bispinosa* (Forssk.) Vahl, *Blepharis ciliaris* (L.) B.L.Burtt and *Hypoestes forskaolii* (Vahl) R.Br. (Acanthaceae); *Aloe officinalis* Forssk. (Aloeaceae), *Aerva javanica* (Burm.f.) Juss. ex Schult., *Aerva lanata* (L.) A. L. Juss. ex Schultes and *Celosia* spp. (Amaranthaceae); *Adenium obesum* (Forssk.) Roem. & Schlt. and *Carissa edulis* (Forssk.) Vahl (Apocynaceae); *Commiphora quadricinta* Schweinf. and *Capparis cartilaginea* Decne. (Burseraceae); *Commelina forskaolii* Vahl (Commelinaceae); *Conyza stricta* Willd., *Echinops* sp., *Psiadia punctulata* (DC.), *Pulicaria undulata* (DC.), *Rhamnus dispermus* (L.), *Tagetes minuta* L. and *Vernonia schimperi* DC. (Compositae); *Sansevieria ehrenbergii* Schweinf. ex Baker (Dracaenaceae); succulent *Euphorbia* spp. (Euphorbiaceae); *Acacia asak* (Forssk.), *Acacia etbaica* Schweinf and *Indigofera spinosa* Forssk. (Fabaceae); *Asparagus africanus* Lam. (Liliaceae); *Hibiscus micranthus* L. and *Hibscus deflersii* Schweinf. ex Cufod. (Malvaceae); *Ficus ingens* (Miq.) (Moraceae); *Commicarpus* spp. (Nyctaginaceae); *Aristida adscensionis* L., *Cenchrus ciliaris* L., *Eragrostis tenella* (L.) P. Beauv. ex Roemer & Schultes and *Pennisetum divisum* (Gmel.) Henr. (Poaceae); *Solanum incanum* L. (Solanaceae); *Grewia tembensis* Fresen and *Grewia tenax* (Forssk.) (Tiliaceae); *Cissus rotundifolius* (Forssk.) Vahl (Vitaceae); in addition to semi-evergreen sclerophyllous woodlands of the Afromontane vegetation (Ghazanfar and Fisher 1998; Zohary 1973; Thomas 2016).

Sclater (1858) and Wallace (1876) proposed the classical zoogeographical regions and placed the northern border of the Afrotropics along the Tropic of Cancer, i.e. the northern limit of the Afrotropical Region was placed in Taif area, some 200 km north of Al-Baha Province (Hölzel 1998). Crosskey (1980) considered the northern boundaries of Yemen as the regional boundary between the Afrotropical and Palaearctic parts in the Arabian Peninsula. Extensive sampling of Insects in the Arabian Peninsula by many authors in Yemen, Oman, the United Arab Emirates and south-western mountains of KSA, have raised some interesting questions about the true extent of the Afrotropical Region in this important transitional zone. Authors indicate that Wallace’s (1876) concept of the extent of the Afrotropical Arabian Peninsula is more accurate than Crosskey’s (1980) limited concept of Yemen alone (Kirk-Spriggs and McGregor 2009, El-Hawagry et al. 2015). However, Uvarov (1938), Greathead (1980) and Larsen (1984) agreed that the south-western part of KSA including the study area should be united with the central Arabian deserts which are either considered as a part of the Palaearctic or by some authors as an autonomous Eremic Zone (also called the Saharo-Sindian faunal region).

## Material and methods

Flies were collected from different localities in SANR over two successive years, 2014 and 2015 by the authors. Twelve collecting trips were made, six in 2014 in February, April, June, August, October and December, and six in 2015 in January, March, May, July, September and November. Collections were made in 6 different localities representing different altitudinal levels and habitats in SANR (Figs 13–18, Table 1). The collecting methods included sweep and aerial nets (randomly), bait traps (irregularly), light traps (6 traps, one in each locality, for one night in each trip), Malaise traps (6 traps, one in each locality, for one day in each trip), pitfall traps (90 traps, 15 in each locality, for three days in each trip), and vacuuming (one time in each locality, for 15 minutes in each trip). In addition, a few specimens were incidentally collected by hand.

**Table 1.** An overview of the collecting localities with their coordinates and common vegetation.

Locality no.	Coordinates (in decimal degrees)			The most common plants in the locality	
	Elevation (M)	Latitude (N)	Longitude (E)	Species	Family
**1**	1666	19.8429	41.3115	*Barleria bispinosa* (Forssk.)	Acanthaceae
				*Carissa edulis* L.	Apocynaceae
				*Conyza stricta* Willd.	Compositae
				*Psiadia punctulata* (DC.)	,,
				*Rhamnus dispermus* (L.)	,,
				*Aristida adscensionis* L.	Poaceae
				*Acacia etbaica* Schweinf	Fabaceae
				*Indigofera spinosa* Forssk.	,,
				*Hibiscus micranthus* L.	Malvaceae
				*Hibscus deflersii* Schweinf. ex Cufod.	,,
**2**	1611	19.8402	41.3114	*Barleria bispinosa* (Forssk.)	Acanthaceae
				*Hypoestes forskaolii* (Vahl) *Aerva javanica* (Burm.f.)	,, Amaranthaceae
				*Capparis cartilaginea* Decne.	Burseraceae
				*Echinops* sp.	Compositae
				*Pulicaria undulata* (DC.)	,,
				*Tagetes minuta* L.	,,
				*Vernonia schimperi* DC.	,,
				*Cenchrus ciliaris* L.	Poaceae
				*Eragrostis tenella* (L.)	,,
				*Pennisetum divisum* (Gmel.)	,,
				*Indigofera spinosa* Forssk.	Fabaceae
				*Ficus ingens* (Miq.)	Moraceae
				*Commicarpus* spp.	Nyctaginaceae
				*Solanum incanum* L.	Solanaceae
**3**	1563	19.8388	41.3101	*Barleria bispinosa* (Forssk.)	Acanthaceae
				*Aerva javanica* (Burm.f.)	Amaranthaceae
				*Aerva lanata* (L.)	,,
				*Asparagus africanus* Lam.	Liliaceae
				*Commiphora quadricinta* Schweinf.	Burseraceae
				*Commelina forskaolii* Vahl	Commelinaceae
				*Tagetes minuta* L.	Compositae
				*Aristida adscensionis* L.	Poaceae
				*Cenchrus ciliaris* L.	,,
				*Eragrostis tenella* (L.)	,,
				*Indigofera spinosa* Forssk.	Fabaceae
				*Solanum incanum* L.	Solanaceae
				*Grewia tembensis* Fresen	Tiliaceae
				*Grewia tenax* (Forssk.)	,,
				*Cissus rotundifolius* (Forssk.)	Vitaceae
**4**	1474	19.8452	41.3044	*Aerva javanica* (Burm.f.)	Amaranthaceae
				*Adenium obesum* (Forssk.)	Apocynaceae
				*Tagetes minuta* L.	Compositae
				*Cenchrus ciliaris* L.	Poaceae
				*Acacia asak* (Forssk.)	Fabaceae
				*Acacia etbaica* Schweinf	,,
				*Indigofera spinosa* Forssk.	,,
				*Solanum incanum* L.	Solanaceae
**5**	1325	19.8511	41.3006	*Barleria bispinosa* (Forssk.)	Acanthaceae
				*Blepharis ciliaris* (L.)	,,
				*Aerva javanica* (Burm.f.)	Amaranthaceae
				*Aerva lanata* (L.)	,,
				*Acacia asak* (Forssk.)	Fabaceae
				*Acacia etbaica* Schweinf	,,
				*Indigofera spinosa* Forssk.	,,
				*Solanum incanum* L.	Solanaceae
**6**	1225	19.8627	41.3015	*Barleria bispinosa* (Forssk.)	Acanthaceae
				*Blepharis ciliaris* (L.)	,,
				*Aloe officinalis* Forssk.	Aloeaceae
				*Psiadia punctulata* (DC.)	Compositae
				*Sansevieria ehrenbergii* Schweinf.	Dracaenaceae
				*Cenchrus ciliaris* L.	Poaceae
				*Acacia asak* (Forssk.)	Fabaceae
				*Solanum incanum* L.	Solanaceae

**Table 2.** Zoogeographic affinities of fly species of Jabal Shada al-A’la Nature Reserve (SANR).

Region	Affinities	
	No. of species	%
Afrotropical	55	46
Palaearctic	28	23.5
Oriental	3	2.5
Cosmopolitan	14	12
Undetermined	19	16

All taxa are identified and arranged in alphabetical order. Dates of collection for each species are included for the purpose of mapping the activity periods of species in the study area. Each collection date is followed, between parentheses, by the method of collection used, and the latter is followed by the locality number from which the specimens are collected.

Zoogeographical affiliations of species reported in the study area were estimated using world catalogues and counted to calculate the percentage of Afrotropical, Palaearctic or Oriental elements.

Images of newly recorded species were made using a Leica MZ 125 stereo-binocular microscope (Leica Microsystems Ltd, St. Gallen, Switzerland) fitted with a digital camera (Q-imaging Micro Publisher 5.0 RTV; Zerene Systems LLC, Richland, WA, USA) at the Plant Protection Department, College of Food and Agriculture Sciences, King Saud University. Photo automontage was performed by Zerene stacker program version 1.04 (Innovative Solutions, Bucharest, Romania).

Many studies and keys have been consulted in order to identify, classify and estimate the zoogeographical affiliation of collected specimens and such studies are indicated after each taxon in the list, in addition to the following: Abdullah and Merdan (1995), Amoudi (1993), Dawah and Abdullah (2006), El-Hawagry (2015), El-Hawagry and Gilbert (2014), El-Hawagry et al. (2000), Evenhuis and Greathead (2015), Greathead (1980, 1988), Londt (2008), McAlpine (1981), Pape (1996), Pape and Thompson (2016), Soós and Papp (1984–1993), Unwin (1991).

Unidentified specimens (or photos of specimens)were sent to experts for identification, as indicated after each of these taxa in the list.

Flies of suborder Nematocera were examined and preserved in alcohol, while other flies were glued to pinned stiff paper points, and all are deposited at the King Saud University Museum of Arthropods, Riyadh, Saudi Arabia (KSMA).

Abbreviations used:

AF
 Afrotropical 


BT
 Bait trap 


HP
 Hand-collecting 


KSMA
 King Saud University Museum of Arthropods, Riyadh, Saudi Arabia 


LT
 Light trap 


MT
 Malaise trap 


NE
 Nearctic 


OR
 Oriental 


PA
 Palaearctic 


PT
 Pitfall trap 


SANR
 Jabal Shada al-A’la Nature Reserve 


SW
 Sweeping and areal nets 


VC
 Vacuuming 


## Results

A total of 119 fly species belonging to 87 genera, 31 tribes, 42 subfamilies, and representing 30 families was recorded from SANR through the present study. Some species have been identified only to genus and listed herein as the genera were not previously recorded from KSA.

Most of the recorded fly species are characteristic of the Afrotropical region. Table (2) indicates the zoogeographic affinities of recorded species suggesting a closer affiliation to the Afrotropical region (46%) than to the Palearctic region (23.5%) or the Oriental region (2.5%).

### List of species recorded at SANR to date

Order **Diptera**

Suborder **Nematocera**

Family **Bibionidae**

*Dilophus tridentatus* Walker, 1848

15 February 2014 (MT1), 5 May 2015 (SW1).

Identification: Haenni (1985).

Known distribution: AF.

Family **Ceratopogonidae**

Subfamily **Ceratopogoninae**

Tribe **Culicoidini**

*Culicoides kingi* (Austen, 1912)

23 August 2014 (LT2, LT5).

Identification: Alahmed et al. (2010), Boorman (1989).

Known distribution: AF.

Subfamily **Forcipomyiinae**

*Forcipomyia sahariensis* Kieffer, 1923

23 August 2014 (LT1).

Identification: Lewanczyk et al. (2009).

Known distribution: AF. First record from KSA.

Family **Culicidae**

Subfamily **Anophelinae**

*Anopheles multicolor* Cambouliu, 1902

23 August 2014 (LT2), 15 February 2014 (LT3).

Identification: Glick (1992).

Known distribution: PA.

Subfamily **Culicinae**

*Aedes caspius* (Pallas, 1771)

15 February 2014 (LT1, PT4).

Identification: Alikhan et al. (2014).

Known distribution: PA.

*Culex pipiens* Linnaeus, 1758

23 August 2014 (PT4).

Identification: Thielman and Hunter (2007).

Known distribution: Cosmopolitan.

Family **Sciaridae**

*Chaetosciara* sp. Fig. 7

15 February 2014 (MT1), 23 August 2014 (LT2).

*Remark*: This seems to be the first record of Sciaridae from KSA..

Identification: Steffan (1981) and Mohrig et al. (2012).

Known distribution: Undetermined.

**Figures 3–7.**
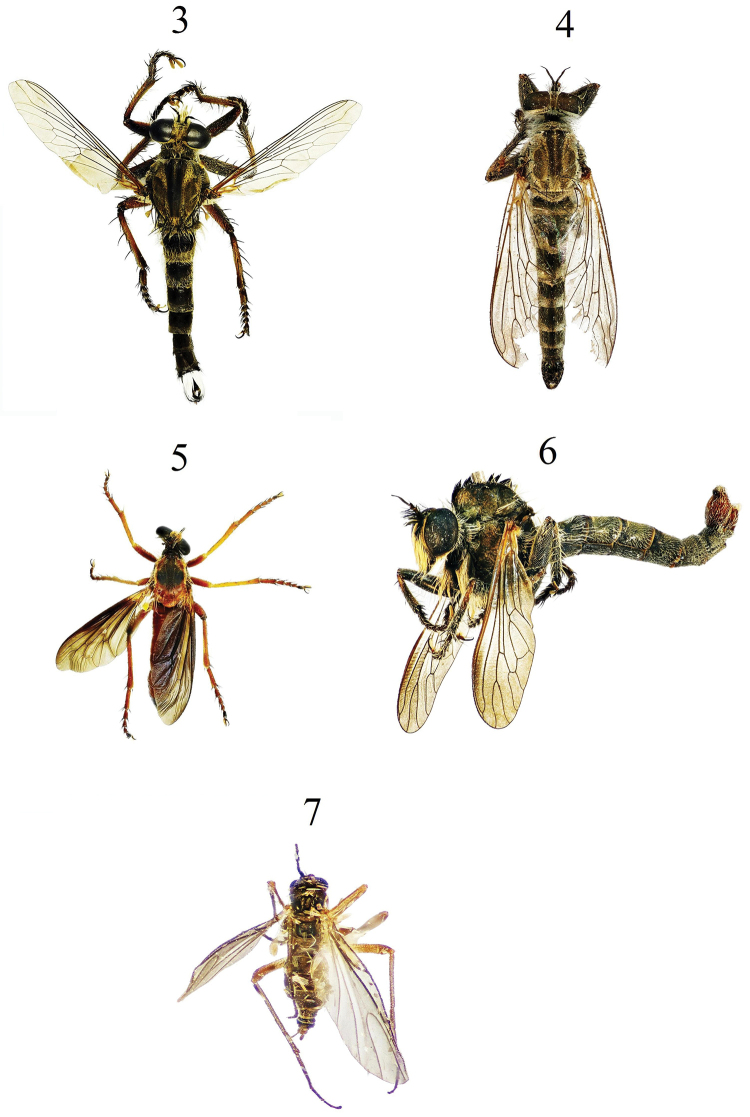
**3** *Promachus sinaiticus* Efflatoun **4** *Neolophonotus* sp.1 **5** *Saropogon longicornis* (Macquart) **6** *Neolophonotus* sp.2 **7** *Chaetosciara* sp.

Suborder **Brachycera**

Infraorder **Asilomorpha**

Superfamily **Asiloidea**

Family **Asilidae**

Subfamily **Asilinae**

Tribe **Asilini**

*Neolophonotus* sp1. Fig. 4

14-15 February 2014 (MT1, MT3), 21 April 2014 (LT3), 27 January 2015 (MT2, MT3, MT5), 5 May 2015 (SW1), 27 July 2015 (LT2).

*Remark*: This seems to be the first record of this genus from KSA.

Identification: Dr. Jason G.H. Londt, from photos (personal communication).

Known distribution: Undetermined.

*Neolophonotus* sp2. Fig. 6

15 February 2014 (MT3), 15 November 2015 (MT3).

*Remark*: This seems to be the first record of this genus from KSA.

Identification: Dr. Jason G.H. Londt, from photos (personal communication).

Known distribution: Undetermined.

Subfamily **Apocleinae**

*Promachus sinaiticus* Efflatoun, 1934 Fig. 3

20 April 2014 (LT6), 3 June 2014 (LT2, MT4), 3-5 June 2014 (SW2), 15 November 2015 (MT6).

Identification: Efflatoun (1934, 1937).

Known distribution: PA. First record of the species from the KSA.

Subfamily **Dasypogoninae**

Tribe **Dasypogonini**

*Saropogon longicornis* (Macquart, 1838) Fig. 5

3 June 2014 (MT3).

Identification: Efflatoun (1934, 1937).

Known distribution: PA. First record from KSA.

*Saropogon* sp.

15 November 2015 (MT6).

*Remark*: This seems to be the first record of this genus from KSA.

Identification: Efflatoun (1934, 1937).

Known distribution: Undetermined.

Subfamily **Laphystiinae**

*Trichardis leucocomus* (Wulp, 1899)

3 June 2014 (MT5), 5 May 2015 (MT5).

Identification: Dr Torsten Dikow, from photos (personal communication).

Known distribution: PA.

Family **Bombyliidae**

Subfamily **Bombyliinae**

Tribe **Bombyliini**

*Bombylella delicata* Wiedemann, 1830

5 June 2014 (SW6), 28 July 2015 (SW3).

Identification: Magdi El-Hawagry using Greathead (1980, 1988).

Known distribution: AF.

*Bombylius pallidipilus* Greathead, 1967

15 February 2014 (MT1), 23 August 2014 (LT2).

Identification: Magdi El-Hawagry using Greathead (1980, 1988).

Known distribution: AF.

*Systoechus horridus* Greathead, 1980

21 April 2014 (LT2), 3 May 2015 (LT5), 14 November 2015 (LT6).

Identification: Magdi El-Hawagry using Greathead (1980, 1988).

Known distribution: PA.

Subfamily **Anthracinae**

Tribe **Anthracini**

*Anthrax sticticus* Klug, 1832

22 April 2015 (LT).

Identification: Magdi El-Hawagry using Greathead (1980, 1988).

Known distribution: AF, PA.

*Spogostylum candidum* (Sack, 1909)

4 June 2014 (SW6).

Identification: Magdi El-Hawagry using Greathead (1980, 1988).

Known distribution: OR, PA.

*Spogostylum isis* (Meigen, 1820)

29 July 2015 (PT5).

Identification: Magdi El-Hawagry using Greathead (1980, 1988).

Known distribution: PA.

*Spogostylum tripunctatum* (Pallas *in* Wiedemann, 1818)

4-5 June 2014 (SW2), 2 September 2015 (LT6).

Identification: Magdi El-Hawagry using Greathead (1980, 1988).

Known distribution: PA. First record from KSA.

Tribe **Exoprosopini**

*Defilippia nigrifimbriata* (Hesse, 1956)

17 October 2014 (MT5).

Identification: Magdi El-Hawagry using Greathead (1980, 1988).

Known distribution: AF.

*Exoprosopa disrupta tihamae* Greathead, 1980 Fig. 9

3 June 2014 (SW1).

Identification: Magdi El-Hawagry using Greathead (1980, 1988).

Known distribution: AF.

**Figures 8–12.**
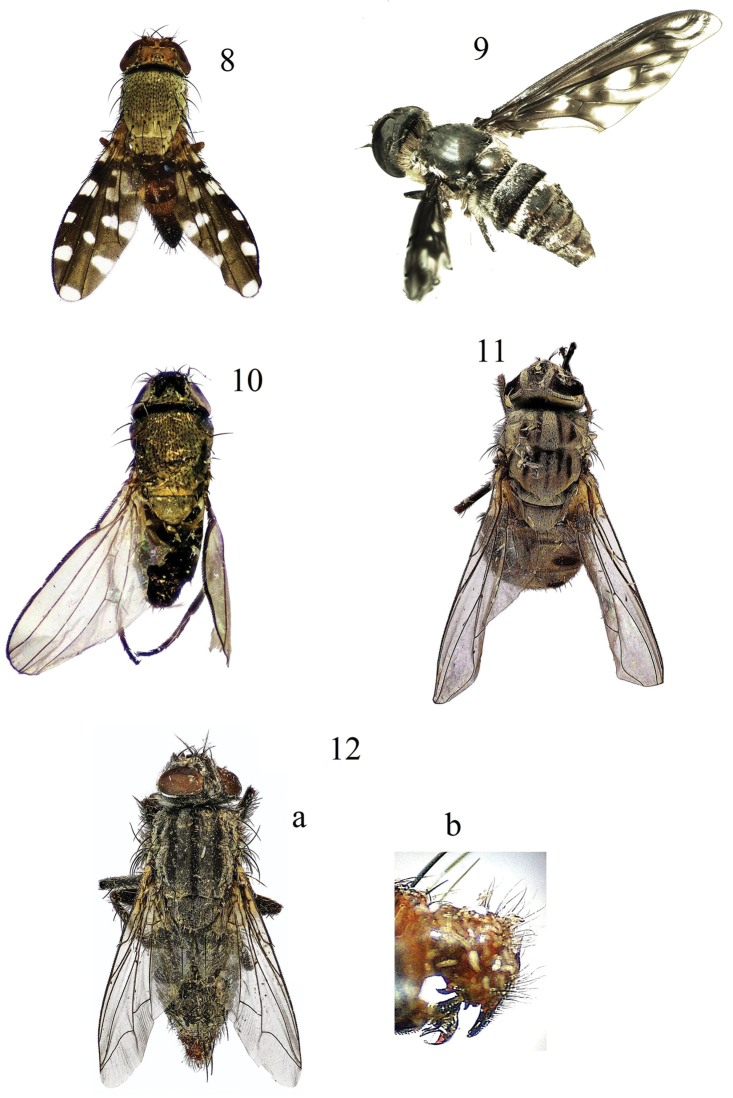
**8** *Actocetor margaritatus* Wiedemann **9** *Exoprosopa disrupta tihamae* Greathead **10** *Desmometopa inaurata* Lamb **11** *Stomoxys niger* Macquart **12 a** *Sarcophaga palestinensis* (Lehrer), habitus **b** same, male genitalia.

**Figures 13–18.**
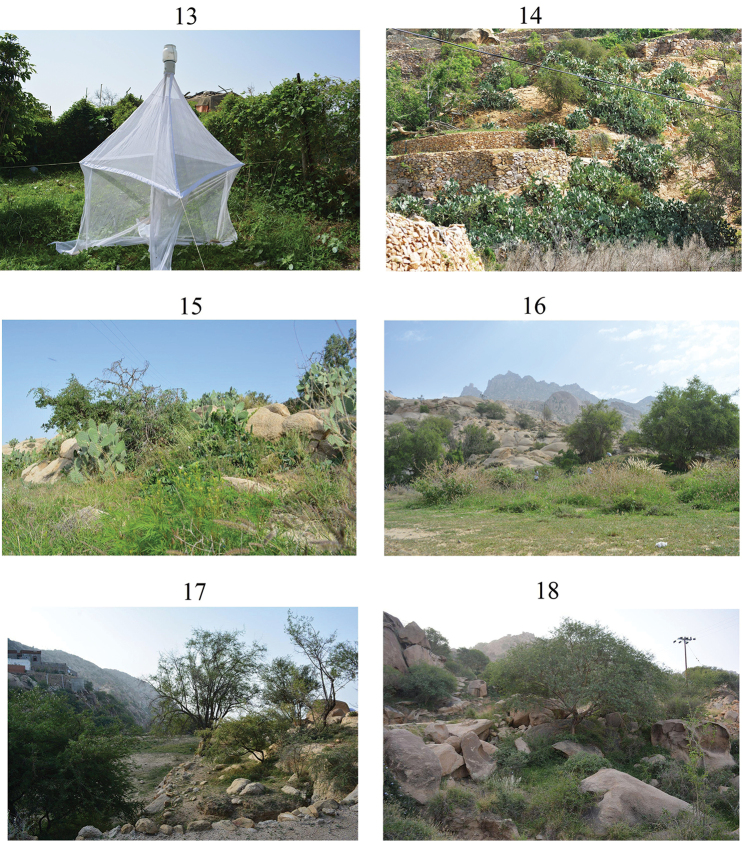
**13** Collecting locality no. 1 **14** Collecting locality no. 2 **15** Collecting locality no. 3 **16** Collecting locality no. 4 **17** Collecting locality no. 5 **18** Collecting locality no. 6.

*Heteralonia bisecta* Greathead, 1988

29 July 2015 (PT5).

Identification: Magdi El-Hawagry using Greathead (1980, 1988).

Known distribution: AF.

*Pterobates chalybaeus* (Röder, 1887)

3 November 2014 (HP6).

Identification: Magdi El-Hawagry using Greathead (1980, 1988).

Known distribution: PA.

Tribe **Villini**

*Exhyalanthrax triangularis* Bezzi, 1924

27 January 2015 (MT5), 5 May 2015 (MT2, MT4), 15 November 2015 (MT4).

Identification: Magdi El-Hawagry using Greathead (1980, 1988).

Known distribution: AF.

*Pachyanthrax circe* (Klug, 1832)

5 May 2015 (MT4).

Identification: Magdi El-Hawagry using Greathead (1980, 1988).

Known distribution: AF.

*Villa bivirgata* Austen, 1937

3 June 2014 (SW4), 5 May 2015 (SW4).

Identification: Magdi El-Hawagry using Greathead (1980, 1988) and EL-Hawagry and Greathead (2006).

Known distribution: PA.

*Villa paniscoides* Bezzi, 1912

3 June 2014 (SW4), 27-28 July 2015 (SW1), 15 November 2015 (MT4).

Identification: Magdi El-Hawagry using Greathead (1980, 1988) and EL-Hawagry and Greathead (2006).

Known distribution: AF.

Tribe **Xeramoebini**

*Desmatoneura* sp.

4 June 2014 (SW4).

Identification: Magdi El-Hawagry using El-Hawagry and Evenhuis (2008).

Known distribution: Undetermined.

*Petrorossia albula* Zaitzev, 1962

5 June 2014 (SW2), 27 July 2015 (SW1).

Identification: Magdi El-Hawagry using Greathead (1980, 1988).

Known distribution: PA.

*Petrorossia letho* (Wiedemann, 1828)

5 June 2014 (SW4), 27 July 2015 (SW1).

Identification: Magdi El-Hawagry using Greathead (1980, 1988).

Known distribution: PA.

*Petrorossia tropicalis* Bezzi, 1921

3-5 June 2014 (SW2, SW4), 5 May 2015 (MT3), 27 July 2015 (SW4).

Identification: Magdi El-Hawagry using Greathead (1980, 1988).

Known distribution: AF.

Family **Therevidae**

*Phycus* sp.

1 June 2014 (LT5), 24 August 2014 (LT6).

*Remark*: This seems to be the first record of the genus from KSA.

Identification: Dr Martin Hauser (personal communication).

Known distribution: AF.

Superfamily **Empidoidea**

Family **Dolichopodidae**

Subfamily **Diaphorinae**

*Asyndetus albifacies* Parent, 1929

27 July 2015 (SW).

Identification: Grichanov (2007).

Known distribution: AF.

Subfamily **Dolichopodinae**

*Dolichopus* sp.

23 August 2014 (LT4), 10 December 2014 (LT6), 26 January 2015 (PT4), 27 July 2015 (LT6).

Identification: Grichanov (2007).

Known distribution: Undetermined.

*Tachytrechus planitarsis* Becker, 1907

23 August 2014 (LT2).

Identification: Grichanov (2007).

Known distribution: PA.

Superfamily **Nemestrinoidea**

Family **Nemestrinidae**

*Trichopsidea costata* Loew, 1858

10 December 2014 (LT6).

Identification: Narchuk (2007).

Known distribution: AF.

Superfamily **Tabanoidea**

Family **Tabanidae**

*Haematopota pluvialis* (Linnaeus, 1758)

15 November 2015 (LT6).

Identification: Amoudi and Leclercq (1992) and Leclercq (1982, 1986, 2000).

Known distribution: PA.

Infraorder **Muscomorpha**

Section **Aschiza**

Superfamily **Platypezoidea**

Family **Phoridae**

*Megaselia scalaris* (Loew, 1866)

23 April 2014 (PT2, PT3), 5 June 2014 (PT4), 2 March 2015 (PT4), 29 July 2015 (PT5), 23 August 2015 (LT3).

Identification: Magdi El-Hawagry.

Known distribution: Cosmopolitan.

Section **Schizophora**

Subsection **Acalyptratae**

Family **Carnidae**

*Hemeromyia* sp. 23 August 2014 (LT1).

*Remark*: This seems to be the first record of the genus from KSA.

Identification: Sabrosky (1987).

Known distribution: Undetermined.

*Meoneura palaestinensis* Hennig, 1937

23 August 2014 (LT1, PT2).

Identification: Papp (1978).

Known distribution: PA.

Family **Chloropidae**

Subfamily **Chloropinae**

*Pachylophus pellucidus* Becker, 1910

24 August 2014 (MT6).

Identification: Deeming and Al-Dhafer (2012).

Known distribution: AF.

*Thaumatomyia notata* (Meigen, 1830)

27 January 2015 (LT1).

Identification: Deeming and Al-Dhafer (2012).

Known distribution: AF, PA.

Subfamily **Oscinellinae**

*Anatrichus pygmaeus* Lamb, 1918

27 July 2015 (VC5).

Identification: Deeming and Al-Dhafer (2012).

Known distribution: AF.

*Aphanotrigonum subfasciellum* Collin, 1949

4 June 2014(SW4), 24 August 2014 (LT6).

Identification: Deeming and Al-Dhafer (2012).

Known distribution: PA.

*Lasiochaeta vulgaris* (Adams, 1905)

15 February 2014 (MT1), 8 December 2014 (VC1, VC4), 5 May 2015 (MT4).

Identification: Deeming and Al-Dhafer (2012).

Known distribution: AF.

*Polyodaspis robusta* (Lamb, 1918)

15 February 2014 (MT1, PT1), 17 October 2014 (LT1), 27 July 2015 (VC2).

Identification: Deeming and Al-Dhafer (2012) for genus, and Lamb (1918) for species.

Known distribution: AF.

*Scoliophthalmus micantipennis* Duda, 1935

5 May 2015 (MT6).

Identification: Identification: Deeming and Al-Dhafer (2012).

Known distribution: AF.

*Scoliophthalmus trapezoides* Becker, 1903

5 May 2015 (MT6).

Identification: Identification: Deeming and Al-Dhafer (2012).

Known distribution: AF.

Subfamily **Siphonellopsinae**

*Apotropina gregalis* (Lamb, 1937)

23 August 2014 (LT5, LT6, PT2, PT3, PT4, PT5, PT6), 17 October 2014 (LT5), 8 December 2014 (VC4), 2-3 March 2015 (PT4, PT5), 17 July 2015 (LT3, MT4), 15 November 2015 (LT6).

Identification: Identification: Deeming and Al-Dhafer (2012).

Known distribution: AF.

Family **Chyromyidae**

Subfamily **Chyromyinae**

*Somatiosoma eremicolum* Ebejer, 2008

15 February 2014 (MT4).

Identification: Ebejer (2008).

Known distribution: AF.

Family **Conopidae**

Subfamily **Myopinae**

Tribe **Zodionini**

*Zodion cinereum* (Fabricius, 1794)

5 May 2015 (MT6).

*Mei & Stuke J-H (2008) has been consulted to identify this species.*

Identification: Mei and Stuke (2008).

Known distribution: PA.

Family **Diopsidae**

*Diopsis apicalis* Dalman, 1817

5 May 2015 (LT2, SW1).

Identification: Dawah and Abdullah (2008).

Known distribution: AF.

*Sphyracephala beccarii* (Rondani, 1873)

2 June 2014 (LT6), 3 June 2014 (LT3, LT4), 3 June 2014 (MT2), 27 January 2015 (LT4), 5 May 2015 (LT1, SW1), 15 November 2015 (LT6).

Identification: Dawah and Abdullah (2008).

Known distribution: AF.

Family **Drosophilidae**

Subfamily **Drosophilinae**

Tribe **Drosophilini**

*Drosophila melanogaster* Meigen, 1830

17-18 October 2014 (LT3, PT2), 8 December 2014 (PT2), 26-27 January 2015 (LT1, MT1, MT2, PT1, PT2), 2 March 2015 (PT1, PT2, PT4).

Identification: Magdi El-Hawagry.

Known distribution: Cosmopolitan.

*Zaprionus indianus* Gupta, 1970

2 March 2014 (PT5), 23 August 2014 (LT2), 18 October 2014 (PT1, PT2, PT4, PT5).

Identification: Amoudi et al. (1991).

Known distribution: OR.

Family **Ephydridae**

Subfamily **Discomyzinae**

Tribe **Discomyzini**

*Actocetor indicus* (Wiedemann 1824)

23 April 2014 (PT4, PT5), 17 October 2014 (LT4).

Identification: Dawah and Abdullah (2006), Becker (1903) and Wiedemann (1824).

Known distribution: AF.

*Actocetor margaritatus* Wiedemann, 1830 Fig. 8

28 February 2014 (PT3), 23 August 2014 (PT1, PT2, PT4, PT5), 10 December (2014 (LT6), 5 May 2015 (LT4, SW1).

Identification: Dawah and Abdullah (2006), Becker (1903) and Wiedemann (1830).

Known distribution: AF.

Tribe **Psilopini**

*Psilopa nilotica* (Becker, 1903)

15 February 2014 (LT2, MT2), 4 June 2014 (SW4), 29 July 2015 (PT4, PT5).

Identification: Dawah and Abdullah (2006), Becker (1903).

Known distribution: AF, PA.

Subfamily **Hydrelliinae**

*Notiphila ignobilis* Loew, 1862

29 July 2015 (MT6).

Identification: Dawah and Abdullah (2006), Becker (1903).

Known distribution: AF.

Family **Lonchaeidae**

Subfamily **Lonchaeinae**

Tribe **Lonchaeini**

*Silba virescens* Macquart, 1851

15 February 2014 (SW6).

Identification: MacGowan and Friedberg (2009).

Known distribution: AF.

Family **Milichiidae**

Subfamily **Madizinae**

*Desmometopa inaurata* Lamb, 1914 Fig. 10

27 January 2015 (LT2), 29 July 2015 (PT4).

Identification: Deeming (1998).

Known distribution: AF. First record from KSA.

*Desmometopa varipalpis* Malloch, 1927

5 May 2015 (PT5), 29 July 2015 (PT6).

Identification: Identification: Deeming (1998).

Known distribution: AF.

Subfamily **Phyllomyzinae**

*Phyllomyza* sp.

15 February 2014 (LT2), 27 July 2015 (LT2).

Identification: Deeming (1998).

Known distribution: Undetermined.

Family **Pyrgotidae**

*Campylocera ferruginea* Macquart, 1843

15 November 2015 (LT6).

Identification: Dr Valery Korneyev, from photos (personal communication).

Known distribution: AF.

*Eupyrgota latipennis* (Walker, 1849)

3 June 2014 (LT2), 14 November 2015 (LT2).

Identification: Dr Valery Korneyev, from photos (personal communication).

Known distribution: AF.

Family **Sphaeroceridae**

*Rachispoda fuscipennis* (Haliday 1833)

15 February 2014 (PT2, PT3), 23 August 2014 (PT6), 18 October 2014 (LT3, PT1, PT2, PT3, PT4), 8-11 December 2014 (LT2, LT3, LT4, VC1, VC2).

Identification: Magdi El-Hawagry, compared with museum specimens.

Known distribution: PA.

Family **Tephritidae**

Subfamily **Dacinae**

Tribe **Dacini**

*Bactrocera zonata* (Saunders, 1842)

23 August 2014 (LT2), 5 May 2015 (SW1), 27 July 2015 (SW1).

Identification: Merz and Dawah (2005) and Efflatoun (1924).

Known distribution: OR.

Subfamily **Tephritinae**

Tribe **Tephritini**

*Acanthiophilus helianthi* (Rossi, 1794)

23 August 2014 (LT2).

Identification: Merz and Dawah (2005) and Efflatoun (1924).

Known distribution: AF, OR, PA.

*Dioxyna sororcula* (Wiedemann, 1830)

15 February 2014 (MT4), 3 June 2014 (MT4), 8 December 2014 (LT5, VC1).

Identification: Merz and Dawah (2005) and Efflatoun (1924).

Known distribution: AF.

*Goniurellia tridens* (Hendel, 1910)

23 August 2014 (LT2).

Identification: Hendel (1910).

Known distribution: PA.

*Trupanea stellata* (Fuesslin, 1775)

3 June 2014 (LT2).

Identification: Merz and Dawah (2005) and Efflatoun (1924).

Known distribution: PA.

Family **Ulidiidae**

Subfamily **Ulidiinae**

Tribe **Ulidiini**

*Physiphora alceae* (Preyssler, 1791)

15 February 2014 (MT1, LT1), 21 April 2014 (LT1), 6 June 2014 (LT1), 23 August 2014 (LT1), 17-18 October 2014 (LT3, PT3), 27 January 2015 (MT1, MT3), 5 May 2015 (LT1), 27 July 2015 (LT1, SW1), 15 November 2015 (LT6, MT4).

Identification: Al Dhafer and El-Hawagry (2016).

Known distribution: Cosmopolitan.

Subsection **Calyptratae**

Family **Anthomyiidae**

Subfamily **Anthomyiinae**

Tribe **Anthomyiini**

*Anthomyia pluvialis* (Linnaeus, 1758)

15 February 2014 (MT1), 27 January 2015 (MT3), 4-5 May 2015 (MT3, SW1), 15 November 2015 (LT5).

Identification: Michelsen (2008).

Known distribution: PA.

Tribe **Hydrophoriini**

*Delia platura* (Meigen, 1826)

15 February 2014 (LT1, LT2, LT3, MT1), 23 August 2014 (LT2), 17 October 2014 (LT1, LT2), 27 January 2015 (LT2, LT3, MT2).

Identification: Meigen (1826).

Known distribution: Cosmopolitan.

Family **Calliphoridae**

Subfamily **Calliphorinae**

*Calliphora croceipalpis* Jaennicke, 1867

15 February 2014 (MT4).

Identification: Setyaningrum and Al Dhafer (2014).

Known distribution: AF.

*Calliphora vicina* (Robineau-Desvoidy, 1830)

3 June 2014 (SW6).

Identification: Setyaningrum and Al Dhafer (2014).

Known distribution: Cosmopolitan.

Subfamily **Chrysomyinae**

*Chrysomya albiceps* (Wiedemann, 1819)

4 June 2014 (SW1), 2 September 2015 (LT6), 15 November (LT3).

Identification: Setyaningrum and Al Dhafer (2014).

Known distribution: AF.

*Chrysomya putoria* (Wiedemann, 1830)

3 June 2014 (SW4).

Identification: Setyaningrum and Al Dhafer (2014).

Known distribution: AF.

*Chrysomya regalis* Robineau-Desvoidy, 1830

15 February 2014 (MT3), 4 June 2014 (MT6), 10 December 2014 (LT6).Identification: Setyaningrum and Al Dhafer (2014).

Known distribution: AF.

Subfamily **Luciliinae**

*Lucilia sericata* (Meigen, 1826)

16 February 2014 (HP6), 21 February 2014 (LT3), 10 December 2014 (LT6).

Identification:

Known distribution: Cosmopolitan.

Subfamily **Polleniinae**

*Pollenia hungarica* Rognes, 1987

17 October 2014 (LT6).

Identification: Setyaningrum and Al Dhafer (2014).

Known distribution: PA.

*Pollenia rudis* (Fabricius, 1794)

17 October 2014 (LT5).

Identification: Setyaningrum and Al Dhafer (2014).

Known distribution: PA.

Family **Muscidae**

Subfamily **Atherigoninae**

Tribe **Atherigonini**

*Atherigona humeralis* Wiedemann, 1830

15 November 2015 (SW5).

Identification: Pont (1991).

Known distribution: AF.

*Atherigona laevigata* (Loew, 1852)

15 February 2014 (MT1), 8 December 2014 (VC4).

Identification: Pont (1991).

Known distribution: AF.

*Atherigona reversura* Villeneuve, 1936

15 February 2014 (MT3), 23 August 2014 (LT2, LT3, LT5), 17 October 2014 (LT4, LT5, MT2, MT4), 5 May 2015 (MT2), 15 November 2015 (MT4), 2 September 2015 (LT6).

Identification: Pont (1991).

Known distribution: OR.

Subfamily **Coenosiinae**

Tribe **Coenosiini**

*Coenosia attenuata* Stein, 1903

15 February 2014 (MT4, PT4), 23 April 2014 (PT1), 23 August 2014 (LT2), 17 October 2014 (LT2, LT4, MT4), 18 October 2014 (PT5), 5 May 2015 (MT4), 15 November 2015 (MT4).

Identification: Pont (1991).

Known distribution: Cosmopolitan.

*Coenosia humilis* Meigen, 1826

5 May 2015 (MT6).

Identification: Pont (1991).

Known distribution: Cosmopolitan.

Tribe **Limnophorini**

*Lispe nivalis* Wiedemann, 1830

15 February 2014 (LT6).

Identification: Pont (1991).

Known distribution: AF.

*Lispe pectinipes* Becker, 1903

23 August 2014 (LT2, LT3), 17 October 2014 (LT5), 5 May 2015 (LT1, MT2), 14-15 November 2015 (LT4, LT5).

Identification: Pont (1991).

Known distribution: PA.

Subfamily **Muscinae**

Tribe **Muscini**

*Musca albina* Wiedemann, 1830

5 May 2015 (MT6).

Identification: Pont (1991).

Known distribution: AF, OR, PA.

*Musca autumnalis* De Geer, 1776

23 August 2014 (LT2), 5 May 2015 (MT2).

Identification: Pont (1991).

Known distribution: Cosmopolitan.

*Musca calleva* Walker, 1849

14 November 2015 (LT4).

Identification: Pont (1991).

Known distribution: AF.

*Musca domestica* Linnaeus, 1758

15 February 2014 (MT5, PT6), 3 June 2014 (MT2, SW6), 23 August 2014 (LT2, LT3), 5 May 2015 (MT6), 2 September 2015 (LT5), 15 November 2015 (LT6).

Identification: Pont (1991).

Known distribution: Cosmopolitan.

*Musca lucidula* (Loew, 1856)

3 June 2014 (MT6).

Identification: Pont (1991).

Known distribution: AF, PA.

*Musca sorbens* Wiedemann, 1830

5 May 2015 (MT1), 15 November 2015 (LT5).

Identification: Pont (1991).

Known distribution: AF.

Tribe **Stomoxyini**

*Stomoxys niger* Macquart, 1851 Fig. 11

15 February 2014 (MT4), 17 October 2014 (LT5).

Identification: Márcia et al. (2012).

Known distribution: AF. First record from KSA.

Subfamily **Phaoniinae**

Tribe **Dichaetomyiini**

*Dichaetomyia luteiventris* (Rondani, 1873)

2 March 2015 (PT5).

Identification: Pont (1991).

Known distribution: AF.

Tribe **Phaoniini**

*Helina coniformis* (Stein *in* Becker, 1903)

15 February 2014 (MT5, PT2), 21 April 2014 (LT2), 17 October 2014 (LT1, LT5, MT1, MT2, MT3, MT4), 27 January 2015 (MT2, MT3), 14-15 November 2015 (LT4, LT5, MT4).

Identification: Pont (1991).

Known distribution: AF.

*Helina lucida* (Stein, 1913)

21 April 2014 (LT5).

Identification: Pont (1991).

Known distribution: AF.

Family **Rhiniidae**

*Cosmina viridis* Townsend, 1917

15-16 February 2014 (MT1, MT3), 17 October 2014 (LT5), 27 January 2015 (LT1, MT3), 4-5 May 2015 (SW4, MT2).

Identification: Setyaningrum and Al Dhafer (2014).

Known distribution: AF.

*Isomyia terminata* (Wiedemann, 1830)

15 February 2014 (MT5, PT5).

Identification: Setyaningrum and Al Dhafer (2014).

Known distribution: AF.

*Rhinia apicalis* (Wiedemann, 1830)

15 February 2014 (MT5), 3 June 2014 (SW4), 17 October 2014 (LT2, LT3, LT5), 14-15 November 2015 (LT4, LT5, LT6).

Identification: Setyaningrum and Al Dhafer (2014).

Known distribution: AF.

Family **Sarcophagidae**

Subfamily **Miltogramminae**

*Taxigramma heteroneura* (Meigen, 1830)

15 February 2014 (MT5), 3 June 2014 (SW4), 27 January 2015 (MT4), 5 May 2015 (MT4, SW1), 27-29 July 2015 (PT5).

Identification: Thomas Pape (personal communication) and the first author.

Known distribution: NE, PA.

Subfamily **Paramacronychiinae**

*Wohlfahrtia erythrocera* Villeneuve, 1910

28 July 2015 (PT6).

Identification: Thomas Pape (personal communication) and the first author.

Known distribution: AF.

*Wohlfahrtia nuba* Wiedemann, 1830

3 May 2015 (PT5).

Identification: Thomas Pape (personal communication) and the first author.

Known distribution: AF.

Subfamily **Sarcophaginae**

*Blaesoxipha algeriensis* (Townsend, 1919)

23 August 2014 (LT5).

Identification: Thomas Pape (personal communication) and the first author.

Known distribution: PA.

*Blaesoxipha rufipes* (Macquart, 1839)

3 June 2014 (SW4).

Identification: Thomas Pape (personal communication) and the first author.

Known distribution: Cosmopolitan.

*Sarcophaga adhamae* (Lehrer & Abou-Zied, 2008)

21 April 2014 (BT6).

Identification: Lehrer and Abou–Zied (2008).

Known distribution: AF.

*Sarcophaga africa* (Wiedemann, 1824)

5 May 2015 (SW4).

Identification: Thomas Pape (personal communication) and the first author.

Known distribution: Cosmopolitan.

*Sarcophaga babiyari* (Lehrer, 1995)

3 June 2014 (LT6).

Identification: Thomas Pape (personal communication) and the first author.

Known distribution: AF.

*Sarcophaga dux* Thompson, 1869

15 February 2014 (MT1).

Identification: Thomas Pape (personal communication) and the first author.

Known distribution: Cosmopolitan.

*Sarcophaga palestinensis* (Lehrer, 1998) Fig. 12

21 February 2014 (LT1).

Identification: Thomas Pape (personal communication) and the first author.

Known distribution: PA.

Family **Tachinidae**

Subfamily **Exoristinae**

Tribe **Eryciini**

*Drino lota* (Meigen, 1824)

15-16 February 2014 (LT6, MT4, MT5, MT6, SW6), 17 October 2014 (LT4, LT5, LT6), 14-15 November 2015 (LT4, LT6).

Identification: Dawah (2011) and Tschorsnig and Herting (1994).

Known distribution: AF, PA.

Tribe **Exoristini**

*Exorista larvarum* (Linnaeus, 1758)

3 June 2014 (SW2, SW4).

Identification: Dawah (2011) and Tschorsnig and Herting (1994).

Known distribution: NE, PA.

Tribe **Goniini**

*Gonia capitata* (De Geer, 1776)

5 May 2015 (MT1).

Identification: Dawah (2011) and Tschorsnig and Herting (1994).

Known distribution: PA.

*Sturmia bella* (Meigen, 1824)

15 February 2014 (MT1), 21 April 2014 (LT1), 3 June 2014 (SW4), 27-30 January 2015 (LT1, LT2, LT3), 27 July 2015 (LT5).

Identification: Dawah (2011) and Tschorsnig and Herting (1994).

Known distribution: OR, PA.

Subfamily **Phasiinae**

Tribe **Cylindromyiini**

*Cylindromyia bicolor* (Olivier, 1812)

7 June 2014 (SW4).

Identification: Dawah (2011), El-Hawagry et al. (2015) and Tschorsnig and Herting (1994).

Known distribution: PA.

Subfamily **Tachininae**

Tribe **Tachinini**

*Dejeania bombylans* (Fabricius, 1798)

10 December 2014 (LT6).

Identification: Dawah (2011) and Tschorsnig and Herting (1994).

Known distribution: AF.

## Discussion

In terms of vegetation and speciation, the south-western part of KSA, including Al-Baha Province, is considered to be the most important part of the country and the Arabian Peninsula in general. Floristically and ecologically, this area is similar to the high altitude mountains of north-eastern and eastern parts of Africa, and like other areas in the south-western part of the Arabian Peninsula, contains montane woodlands and evergreen shrub lands, with strong Afromontane affinities (Bussmann and Beck 1995; Zohary 1973; Eig 1938).

Considering the insect fauna as a whole, El-Hawagry et al. (2013, 2015) attributed the extraordinary complex and the interesting insect fauna in Al-Baha Province to its geographical position at the junction of two of the world’s main zoogeographical regions, the Afrotropical and the Palaearctic.

Many present day biogeographers think that the biogeographical divisions within the eastern and the northeastern parts of Africa should be extended towards east within the Arabian Peninsula as well, covering the high altitude regions of the southern Al-Sarawat Mountains, namely “Afromontane Archipelago” (Zohary 1973; Eig 1938).

Bolton (1994), Eig (1938), El-Hawagry et al. (2013 and 2015) and Sharaf et al. (2012a, 2012b) concluded that the insect faunal composition in Al-Baha Province has an Afrotropical flavor as the Afrotropical elements were predominantly indicated, they tended to agree with those biogeographers who think that parts of the Arabian Peninsula, including Al-Baha Province, should be included in the Afrotropical region, but they couldn’t indicate the northern border of this region exactly. All these facts seem to be reflected somehow on the fly faunal composition in Jabal Shada al-A’la Nature Reserve (SANR) as shown in the present results which obviously emphasize the fact that Al-Baha Province, as lying in the south-western part of the Arabian Peninsula, should be included in the Afrotropical Region rather than in the Palaearctic Region or the Eremic Zone.

## Funding

The authors would like to extend their sincere appreciation to the Deanship of Scientific Research at King Saud University for its funding this research group NO (RGP-1437-009).
